# Endoplasmic reticulum stress of astrocytes in paraventricular nucleus of hypothalamus promotes ventricular electrical instability after acute myocardial infarction in rats

**DOI:** 10.3389/fcvm.2025.1574146

**Published:** 2025-07-07

**Authors:** Yuhang Yang, Xinmiao Huang, Wenlong Wang, Jingmei Sun, Xin Wang, Chunrui Ji, Xiufen Qu, Dechun Yin

**Affiliations:** ^1^ Department of Cardiology, the First Affiliated Hospital of Harbin Medical University, Harbin, China; ^2^ Department of Cardiology, the First Hospital of Harbin, Harbin, China

**Keywords:** endoplasmic reticulum stress, astrocytes, paraventricular nucleus of hypothalamus, acute myocardial infarction, neuroinflammation, electrophysiology of heart

## Abstract

**Introduction:**

Astrocytes in paraventricular nucleus of hypothalamus (PVN) promote the occurrence of ventricular arrhythmia (VA) after acute myocardial infarction (AMI). However, the mechanism is unclear. The purpose of this study was to investigate the changes of astrocytes in the PVN after AMI, which is involved in ventricular electrical instability in rats.

**Methods:**

The rats were randomly divided into 4 groups: sham operation (SH), AMI, AMI + Vehicle (4 μl for each), AMI + GSK2606414(PERK phosphorylation inhibitor, 90 μg/4 μl for each). PVN was administered by microinjection.

**Results:**

After 24 h, the AMI and AMI + Vehicle groups had substantially greater levels of hypothalamic astrocyte activation, endoplasmic reticulum (ER) stress, and central inflammation (TNF-α and IL-6) compared to the SH group (*P* < 0.05). GSK2606414 microinjection in hypothalamus had no significant effect on glial fibrillary acidic protein (GFAP) positive cells and their pathologic morphology in PVN of AMI + Vehicle group, but significantly reduced ER stress (PERK/CHOP) in the group, alleviating central inflammation and activation of central neurons (*P* < 0.05). Cytological studies confirmed this.

**Conclusion:**

As a result, 24 h after AMI, PVN astrocytes underwent ER stress via PERK/CHOP pathway, which caused central inflammation, sympathetic neuron activation, and increased ventricular electrical activity instability. GSK2606414 microinjection into hypothalamus decreased sympathetic nerve excitement and VA occurrence in AMI rats by inhibiting ER stress of PVN astrocytes.

## 1 Introduction

AMI is associated with a markedly greater incidence of malignant VA, and there is a strong correlation in the development of VA and sudden cardiac death (SCD) due to autonomic nervous system imbalance (1). The occurrence of VA often requires a combination of abnormal structure (matrix) and acute trigger. More and more evidences show that the activation of the sympathetic nervous system is an important contributor to the occurrence of VA and plays a crucial role in both triggering and maintaining VA (2, 3).

The central nervous system (CNS) regulates the electrophysiological substrates that produce VA by modulating autonomic activity. The management of autonomic nerves has been focused on as a means to prevent and manage arrhythmias. However, VA often occur in stroke patients or those patients without cardiac disease, suggesting the significant involvement of the CNS (4, 5). The PVN plays a key role in the maintenance of cardiovascular activities. It directly connects with preganglionic neurons of the sympathetic nervous system and participates in the regulation of peripheral sympathetic nerve activity. Moreover, sensory afferent fibers are also integrated at the PVN level to influence sympathetic nerve emission (6, 7). As a key autonomic nucleus controlling cardiovascular function, PVN plays a crucial role in neuroimmune interactions (8).

Astrocytes are a kind of glial cells responsible for maintaining the proper function of the CNS. Astrocytes can respond to injury, promote neurotoxicity, and mediate inflammation of the CNS by promoting microglia activation and white blood cell transport (9, 10). External stimulation of the brain causes astrocyte activation and increased production of GFAP, a specific marker of astrocytes. Various types of neuroinflammation or cytokines (TNF-α, IL-6, etc.) can regulate the expression of GFAP in different ways. Astrocytes are now regarded as key effectors and propagators of immune signaling, and their role is not merely as support cells, but as active participants in CNS immune responses (11). Additionally, neuronal activity can also regulate GFAP expression (12).

At the early stage of AMI (30 min after coronary artery occlusion), inflammatory cytokines in the PVN begin to increase significantly. However, the exact production and mechanism of these inflammatory cytokines remain unclear (13). Previous studies published by our team have shown that astrocytes expressing the inflammatory protein GFAP play a role in both neuroinflammation in the PVN and the promotion of neuronal activity (14). This may serve as a potential mechanism for the excitation of sympathetic nerves in the CNS during the early stage of AMI. Astrocyte-mediated neuroinflammation may have a significant impact on the early onset of VA in AMI.

Neurological diseases are highly heterogeneous, but generally share a common feature of cellular stress caused by the accumulation of misfolded proteins. Abnormal aggregation of misfolded proteins triggers ER stress while initiating a highly conserved adaptive mechanism called the unfolded protein response (UPR). Despite the extensive research conducted on cells from various tissues, there are still significant differences in the studies of ER stress and UPR pathway in specific cell types under different pathological conditions. Therefore, the purpose of this study was to investigate ER stress of astrocytes in PVN, involved in ventricular electrical instability after AMI in rats and its mechanism.

## 2 Materials and methods

### 2.1 Animals

Male Sprague-Dawley rats (weight 298 g ± 15 g) were purchased from Liaoning Changsheng Biotechnology, China. Every animal was kept in a climate-controlled environment in cages with two rates per cage, a regulated light-dark cycle of 12 h, and free access to food and water. All experiments were approved by the institutional animal care and use Committee of Harbin Medical University and performed according to the “guiding principles for research involving animals and human beings”. All rates were adapted to new environment for one week before experiment.

The rates were randomly divided into 4 groups: SH (*n* = 9), AMI (*n* = 9), AMI + Vehicle (*n* = 9) and AMI + GSK2606414 (*n* = 9). The GSK2606414 or vehicle was injected into the PVN. The doses of GSK2606414 were recommended based on earlier research on this perk blocker. We administered PVN microinjection to rats 2 h prior to AMI surgery. At 24 h after AMI, cardiac electrophysiological data were recorded, then blood and tissues were collected for histopathology, ELISA and Western blot analysis.

### 2.2 The creation of AMI model

AMI model was established by permanent ligation of the left anterior descending coronary artery (LAD). Briefly, each rat was anesthetized by intraperitoneal injection of a mixture of α-chloralose (40 mg/kg) and urethane (800 mg/kg), then each rat was intubated and mechanically ventilated using a rodent ventilator. The heart was exposed via left thoracotomy and LAD was permanently ligated between the left auricle and the pulmonary artery cone. All rats were placed on a heating pad at 37°C to maintain their body temperature until they woke up and then were put back the cages. Bupivacaine (1 mg/kg) was administered at the intercostal incision during closure, and penicillin (0.8 million units) was injected intramuscularly to prevent infection. The electrocardiogram (ECG) was continuously monitored using a computer-based lab system (GY-6328, Huanan Inc., China). The SH animals received same procedure but not LAD ligation.

### 2.3 PVN microinjection

The rats in AMI + GSK2606414 groups were placed in the stereotaxic instrument (kw-dsy, Kew, China) under anesthesia. Based on the Paxinos and Watson rat Atlas, the locations for bilateral PVN were ascertained as follows: 1.8 mm caudal from the bregma, 0.4 mm lateral to the midline, and 7.9 mm vascular to the dorsal surface. The Microregulator needle was inserted into PVN under sterilization. Animals were allowed to recover from the surgery for 7 days. The prepared reagents were injected bilaterally using a microinjector at a dose of 2 μl each side into PVN (90 μg/4 μl for each rat). The bilateral PVN microinjections were completed within 10 min and then removed slowly. At the end of the experiment, Evans blue dye (2%) was injected into the microinjection site for historical verification. For data analysis, only the data from rats whose microinjection sites were inside the borders of the PVN were used. The rats in AMI + Vehicle groups were used the same method (4 μl for each rat).

### 2.4 Electrical study *in vivo*

According to the guidelines provided by the Lambeth conventions for the study of animal VA, the number of VA within 24 h after AMI were continuously recorded, and VA were classified as ventricular fibrillation (VF), ventricular tachycardia (VT) (≥4 conservative VPB), and vascular premature beats (VPB). In our study, spontaneous VA was observed after 24 h. Death rates were separately analyzed. The number of VPB contained isolated VPB, bigeminy and salvo. Furthermore, due to the depth of distinction between VT and VF, they were labeled together. At the end of the experiment, multielectrode catheters (2 mm between electrodes) were placed at the influence border area (IBA, around left vascular base) and influence remote area (IRA, around left vascular apex). The effective refinery period (ERP) is an important index to evaluate the stability of myocardial electrical activity and predict the risk of arrhythmia in electrophysiological examination, which were measured by programmed electrical stimulation comparing an 8-beat drive train (S1, 120 ms cycle length) followed by an extra stimulus (S2), and then repeated this procedure with progressive shorter S1–S2 intervals (from 100 ms to vascular ERP. Ventricular ERP was defined as the longest S1–S2 interval. Next, VF threshold (VFT), the minimum voltage to induce sustained VF, was determined in the rates without spontaneous VF. Until VF emerged, the right ventricular apex was repeatedly stimulated with a 20 ms S1-S1 stimulus, increasing in strength by 0.5 V each time.

### 2.5 Western blot

The PVN tissues were observed using a micropunch technique. Tissues were lysed with lysate containing PMSF and protein phosphatase inhibitor. The total protein concentration was determined using BCA protein quantitative Kit (kgp903; keygen biotech, China). Polyvinylidene fluoride (PVDF) membrane is a commonly used solid phase support for the transfer and fixation of proteins isolated from gels, which were included with primary antibody including Rabbit anti GFAP polyclonal antibody (bs-0199r; BIOSs, China, 1:400), Rabbit anti Fos monoclonal antibody (1:1000), rabbit anti-C3 (1:1000), Rabbit anti perk (1:1000), rabbit anti-p-perk (1:1000), rabbit anti-grp78 (1:1000), Rabbit anti chop (1:1000), Rabbit anti TNF-α (1:1000) and rabbit Anti-IL-6 (1:1000); β- Tubulin (1:4000), GAPDH (1:3000) was used as the internal control.

### 2.6 ELISA analysis

According to the manufacturer's instructions, the plasma and PVN tissues were separately measured with commercial rat norepinephrine (NA) kit (Cusabio biotech Co, China).

### 2.7 Histopathology studies

5 μM-thick slices of cardiac tissue were performed according to standard HE trichrome stay procedures. Brain embedded in paraffin was cut into 4 μM-slices for immunohistochemistry. The slices were left overnight at 4°C with primary antibodies: Rabbit anti GFAP polyclonal antibody (Abcam, 1:400) and Rabbit anti FOS monoclonal antibody (c-Fos; 1:50), then dab substrate chromogen system was used for mounting. In three conservative sections, about 1.80 mm from the bregma, the positive cells inside the bilateral borders of the PVN were analyzed for every animal.

### 2.8 *in vitro* cytological experiments

Astrocytes passage culture: The purchased astrocyte cell line was taken and observed under a microscope. And the cell passage operation was performed, when the cell fusion of astrocytes in the culture bottle reached about 80%. Thapsigargin is an ER calcium ATPase (SERCA) inhibitor, which was used to induce ER stress in astrocytes. Determine the effective concentration of Thapsigargin: different concentrations of Thaps are given (0 μM, 0.01 μM, 0.1 μM, 1.0 μM), when the degree of cell fusion reaches about 60%. Before Thaps (1.0 μM) were added 2 h, the GSK2606414 was added to the astrocyte culture mediums and the final concentrations were determined to be 0.01 μM, 0.1 μM and 1.0 μM. Cells in each group were cultured in cell incubator for 24 h, and then proteins of cells were extracted.

### 2.9 Statistical analysis

The SPSS 27.0 software (IBM Corp, USA) was used for statistical analysis. Data were presented as the average ± standard deviation. Comparisons among continuous data were performed using ANOVA followed by SNK-q test. Values of *P* < 0.05 were considered statistically significant.

## 3 Results

### 3.1 General results and transcriptome sequencing

**Figure 1:**
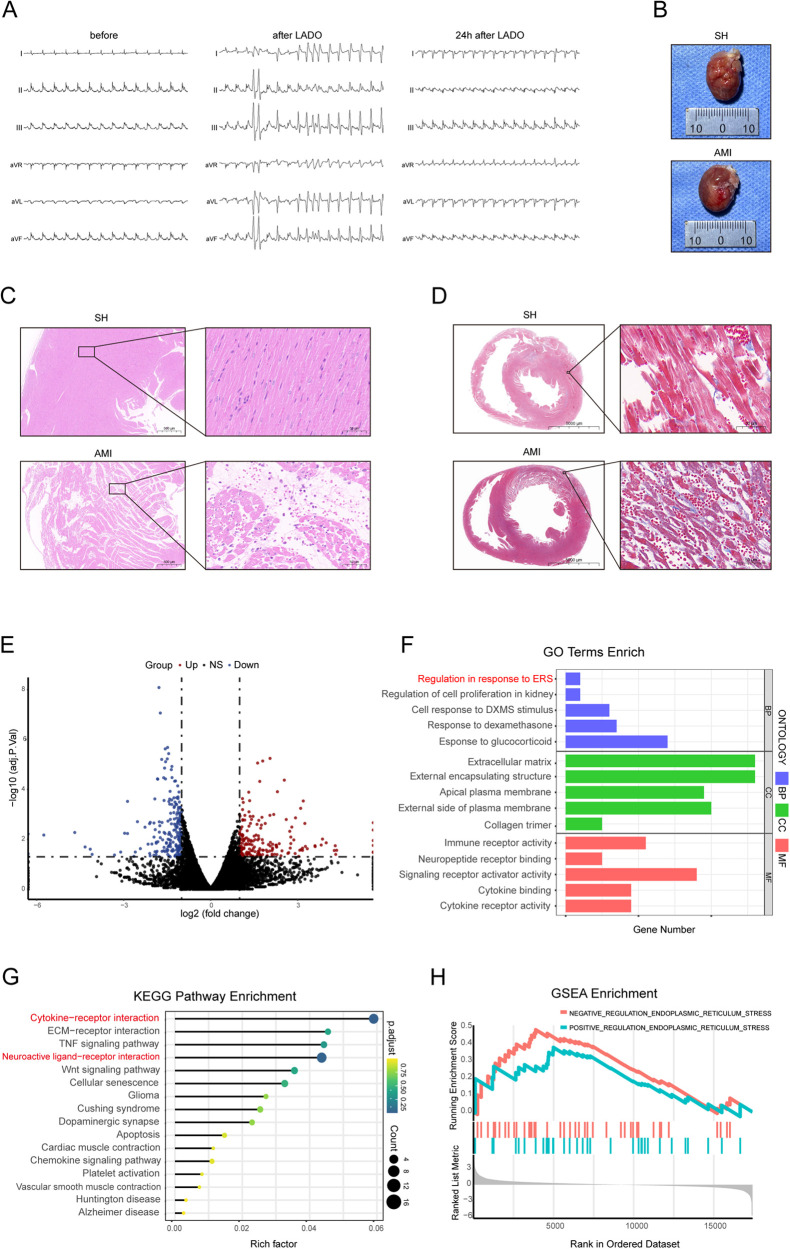
General results and transcriptomic analysis results. **(A)** Changes of body surface ECG prior to, during, and 24 h following LADO; **(B)** Heart tissue of rats in SH group and AMI group; **(C,D)** Cardiac pathology of rats with AMI (scale = 500 μm, 50 μm); **(E)** Gene volcano map of differential expression of PVN tissue; **(F)** GO functional enrichment analysis of differentially expressed genes; **(G)** KEGG functional analysis showed 16 pathways with significant differential expression; **(H)** GSEA enrichment analysis showed that the difference of ER stress genes was most significantly increased. LADO, occlusion of left anterior descending coronary artery; SH, sham operation; AMI, acute myocardial infarction.

Before and after the left anterior descending coronary artery occlusion (LADO) in rats was blocked, dynamic changes in the ECG (ST segment elevation in multiple limb leads followed by Q waves) were observed, along with VA (Figure 1A); The gross heart specimen suggested that the ventricle tissue surface below the ligature was white, and the HE staining of the ventricle showed myocardial cell necrosis and acute inflammatory changes, as well as Masson staining of inflammatory infarct area (Figures 1B–D). These findings suggested that the rat model of AMI was successful.

According to the previous data of the research group, the PVN tissues of five rats each from AMI and sham surgery were used for transcriptome sequencing (RNA SEQ). The RNA SEQ data had been stored in the NCBI database sequence read Archive (SRA) with the accession number of srp193705. The results of Go functional enrichment analysis of differentially expressed genes showed that the significantly enriched items of these genes were related to neuropeptide hormone activity, positive regulation of ER stress, and regulation of cytokines (Figure 1F); Half of the significantly enriched KEGG pathways are related to the nerve and immune system, among which cytokine-cytokine receptor interaction and neuroactive ligand-receptor interaction signaling pathways are more important metabolic pathways and signaling pathways (Figure 1G). The ER stress-related genes in the PVN tissue of AMI rats were overexpressed as compared to the group that had a SH, according to a GSEA analysis of two groups of samples (Figure 1H).

### 3.2 Activation phenotype and neuronal activity of astrocytes in the PVN

**Figure 2:**
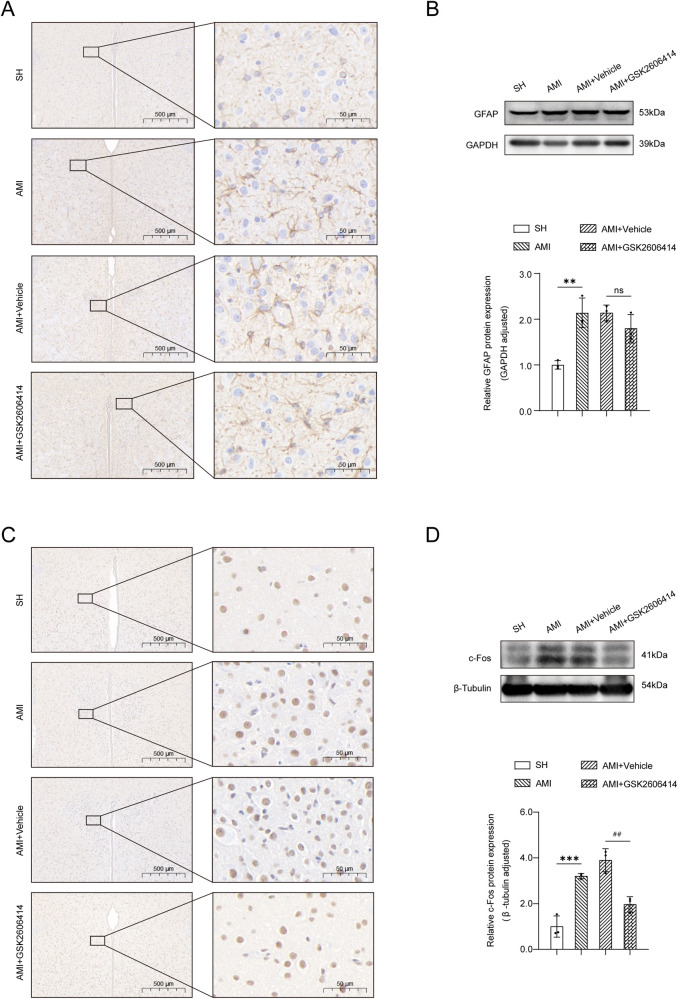
Immunohistochemical results and western blot analysis of the PVN. **(A)** Under a high magnification microscope (scale = 500 μm, 100 μm, 50 μm), the immunohistochemical results of the PVN in the four groups of rats showed that the GFAP immunopositive reaction. **(B)** The expression of GFAP protein in bilateral PVN was determined by western blot. **(C)** Immunohistochemical results of the PVN in the four groups showed that the number of c-Fos positive cells in AMI and AMI + Vehicle rats was higher than that in SH rats, as shown in the representative images of c-Fos immunopositive reaction under high magnification microscope (scale = 500 μm, 50 μm); **(D)** The expression of c-Fos protein in bilateral PVN was determined by western blot. SH, sham operation; AMI, acute myocardial infarction; GSK2606414: p-PERK inhibitor; GFAP, glial fibrillary acidic protein; Compared with SH group, *P < 0.05, **P < 0.01, *n* = 3.

Within 24 h following an AMI, astrocytes inside the PVN exhibited an active morphology, characterized by increased branching and hypertrophy of the cell body (Figure 2A); c-Fos positive cells were significantly increased (Figure 2C). GSK2606414 had no effect on the number of GFAP^+^ cells and their branches in the bilateral PVN in rats, but c-Fos positive cells were significantly reduced. Protein immunoblot analysis also showed the same results (Figures 2B,D).

### 3.3 Paraventricular ER stress and inflammatory cytokine expression in the hypothalamus AMI

At the early stage of AMI (24 h), astrocytes transformed into “A1” phenotype (marker protein C3^+^), which was previously considered to be a pro-inflammatory phenotype with neurotoxicity.

**Figure 3:**
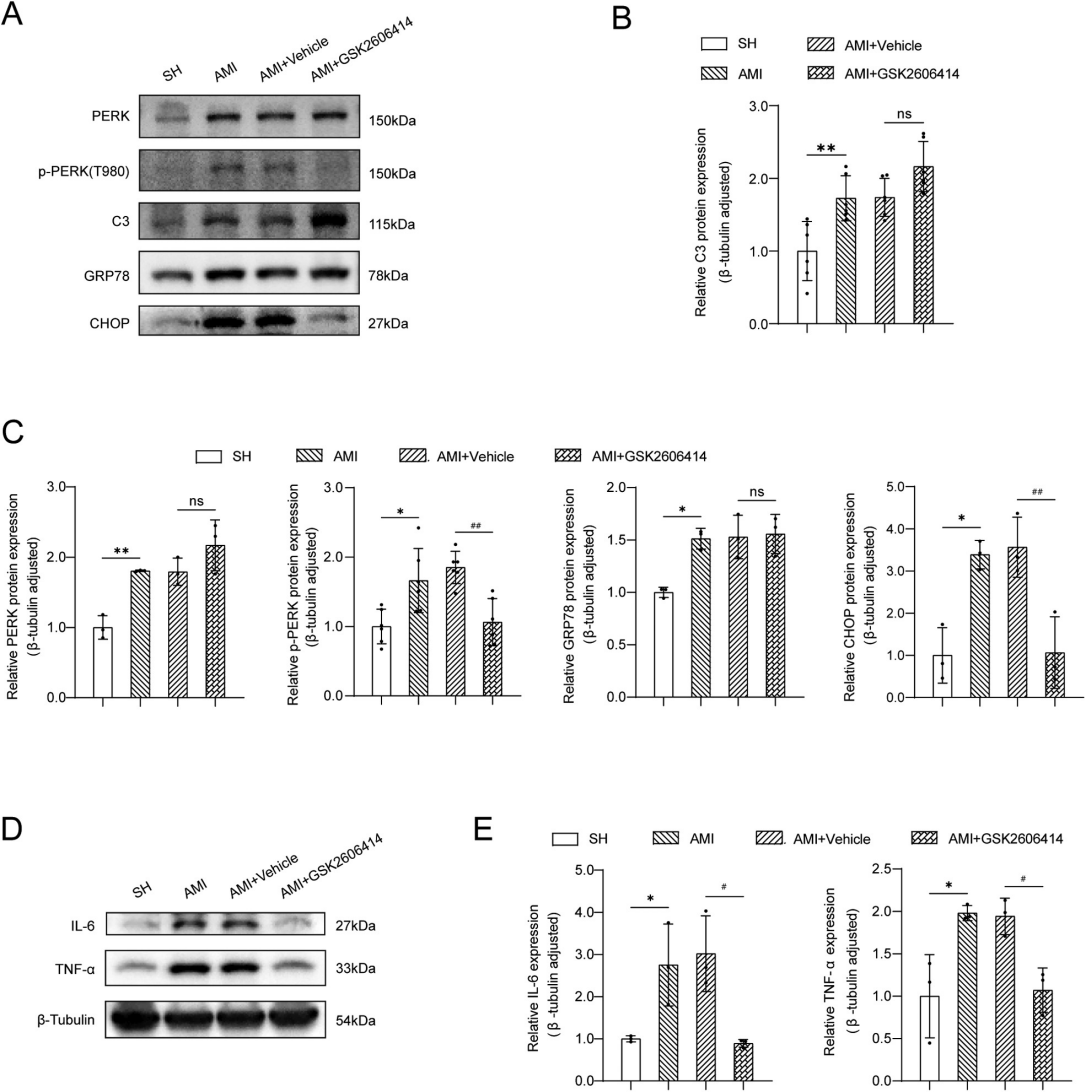
Western blot analysis of ER stress-related proteins and inflammatory cytokines expression in the PVN. **(A)** Western blot results; **(B)** The expression of C3 protein in bilateral PVN was measured by western blot. SH: sham operation; **(C)** The expressions of GRP78, CHOP, PERK and p-PERK in the PVN were measured by western blot; **(D,E)** Expression of TNF-α and IL-6 protein. SH, sham operation; AMI, acute myocardial infarction; GSK2606414: p-PERK inhibitor; Compared with SH group, *P < 0.05, **P < 0.01, *n* = 3.

Compared with the SH group, the expression of ER stress-related proteins (PERK, p-PERK, GRP78, CHOP) was markedly elevated in the early phase following MI, alongside significant increases in TNF-α and IL-6 protein expression levels. GSK2606414 significantly inhibited the protein expression of p-PERK and CHOP in the PVN in rats and reduced the expression of inflammatory cytokines (Figure 3).

### 3.4 Electrophysiological measurement

**Figure 4:**
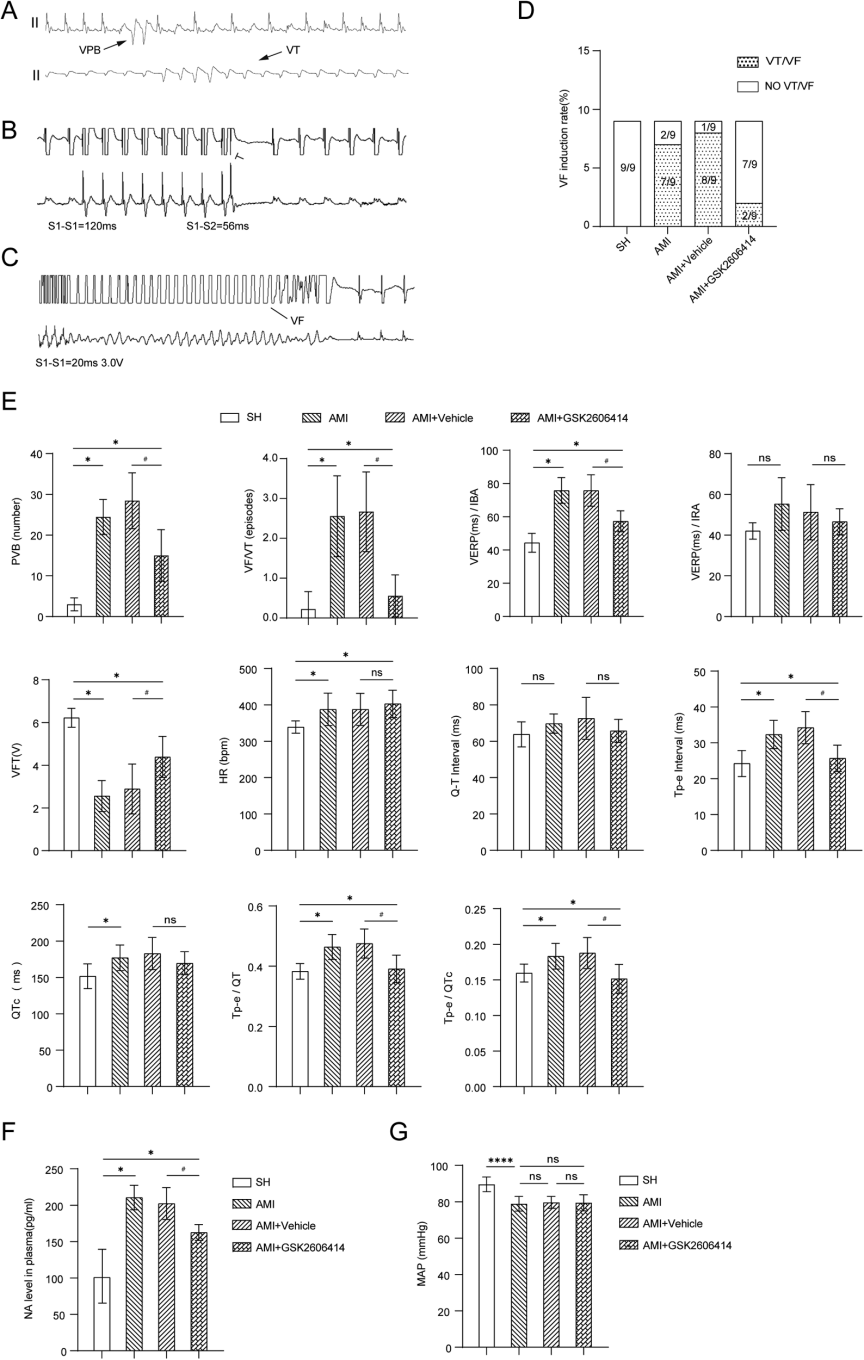
Results of intracardiac electrophysiological testing in four groups of rats. **(A)** ECG findings of VAs; **(B)** Comparison of the programmed stimulation maps of VERP and ERP of left ventricular IBA and IRA in each group of rats; **(C)** The programmed stimulus map of VFT and the comparison of VFT results; **(D)** VFI in each group of rats; **(E)** QT interval, Tp-e interval and QTc of rats in four groups; Influence of Tp-e/QT and Tp-e/QTc ratio; **(F)** The changes of plasma NA in four groups of rats; **(G)** There was no significant difference in mean arterial pressure changes in the four groups of rats. Compared with SH group, *P < 0.05; Compared with AMI + Vehicle group, ^#^*P* < 0.05. SH, sham operation; AMI, acute myocardial infarction; GSK2606414: p-PERK inhibitor; NA, norepinephrine; MAP, marterial pressure; IBA, periinfarction area; IRA, infarct distal region; VERP ventricle, effective refractory period; VFT, ventricular fibrillation threshold; VF, ventricular fibrillation; VFI, ventricular fibrillation induction rate.

In the early stage of our research group, we discovered an interesting observation regarding the inhibition of astrocyte activation through PVN microinjection. We observed a notable difference in the incidence of spontaneous premature ventricular contractions and the prevalence of ventricular tachycardia/ventricular fibrillation between the AMI + GSK2606414 group and the AMI + Vehicle group. This difference was particularly evident within a 24 h (12). Therefore, in this experiment, the ventricular electrophysiology was performed 24 h after AMI in rats. GSK2606414 was found to improve ventricular electrical stability after AMI (Figure 4).

### 3.5 Effects of thaps on astrocytes

**Figure 5:**
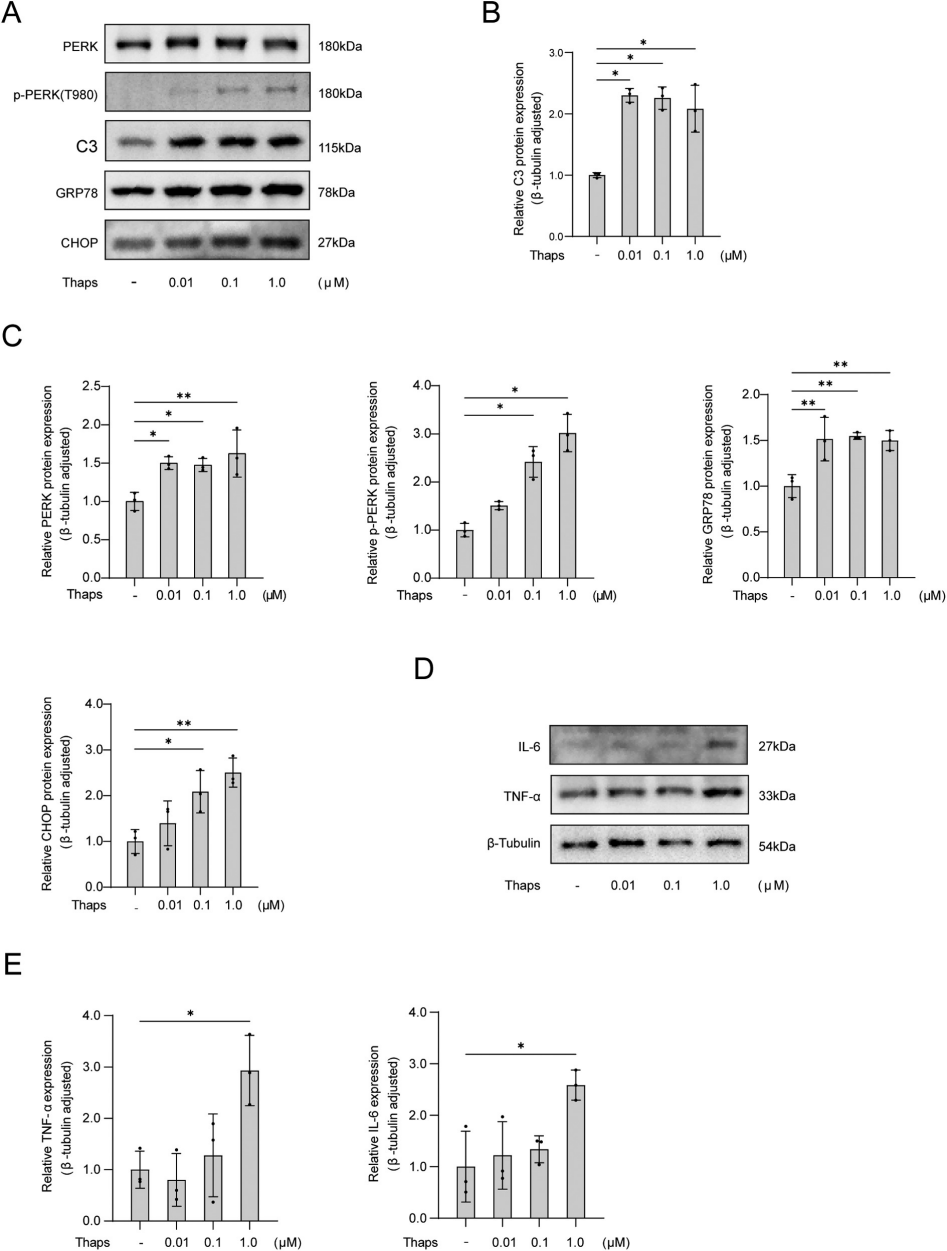
Western blot analysis of thaps on astrocytes. **(A)** Western blot results; **(B)** The expression of C3 protein in astrocytes was observed with an increased concentration of Thaps; **(C)** Expression of proteins associated with UPR initiated by ER stress in astrocytes under conditions of increased Thaps concentration; **(D,E)** Regulation of the expression of inflammatory factor protein in astrocytes by different Thaps concentrations. Compared with Thaps = 0 μM, *P < 0.05, *n* = 3.

Astrocyte cell lines were treated with the indicated Thapsigargin (Thaps) concentrations (0 μM, 0.01 μM, 0.1 μM, 1.0 μM). After a 24 h treatment with Thaps, astrocytes underwent cell protein extraction and Western blot analysis. The findings indicated that astrocytes transformed into pro-inflammatory phenotypes at a concentration of 1.0 μM of Thaps, and the PERK ER stress pathway was triggered (Figure 5).

**Figure 6:**
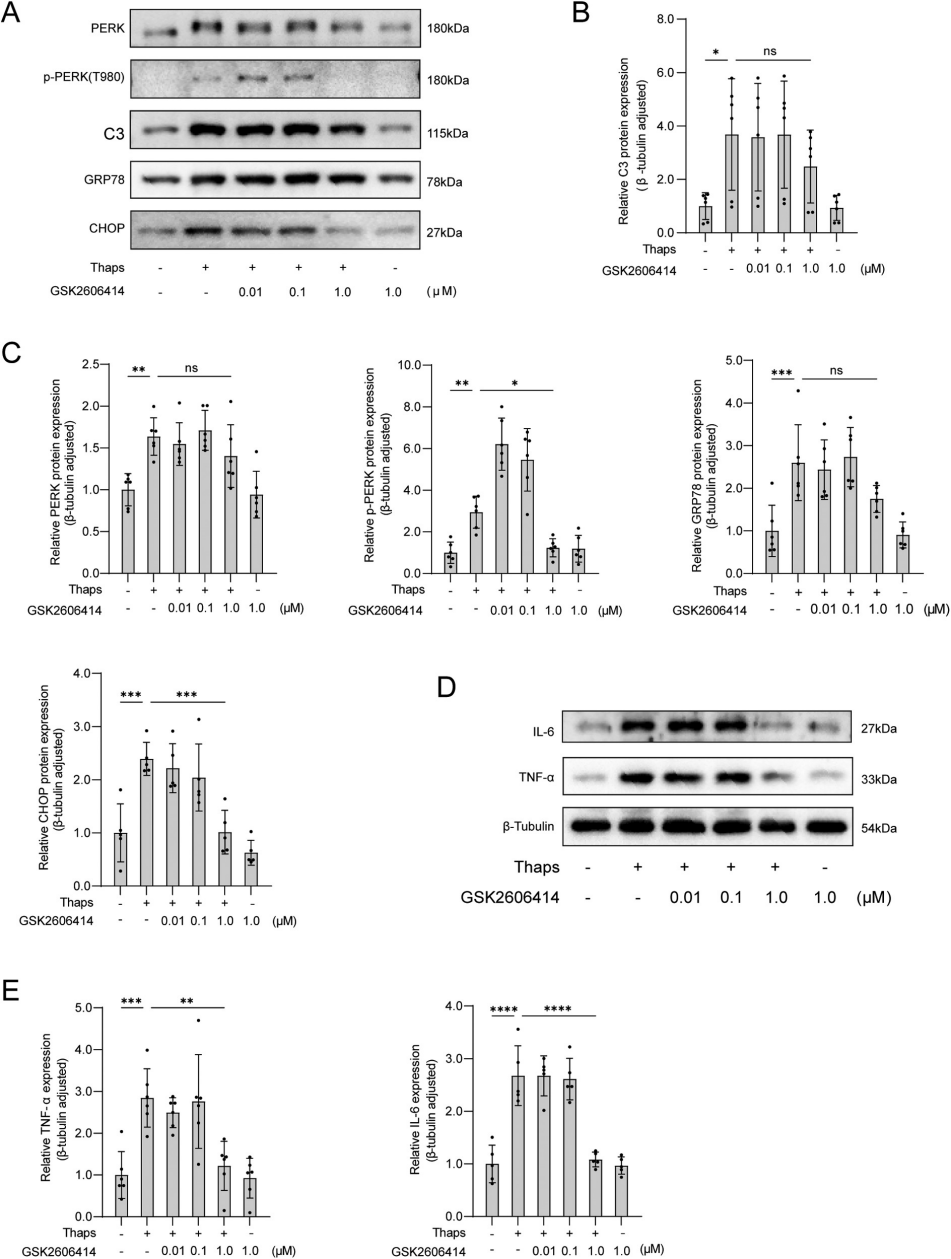
Western blot analysis of the effect of GSK2606414 on thaps-induced astrocytes. **(A)** Western blot results; **(B)** Expression of C3 protein in astrocytes at increased concentration of PERK inhibitor GSK2606414; **(C)** Under conditions of increased concentration of PERK inhibitor GSK2606414, astrocytes were treated with Thaps (1 μM) for 24 h and then western blot was performed; results of TNF-α and IL-6 protein western blot analysis. Compared with Thaps = 0 μM/GSK2606414 = 0 μM, *P < 0.05, compared with Thaps = 1.0 μM/GSK2606414 = 0 μM, ^#^*P* < 0.05, *n* = 6.

### 3.6 Effects of GSK2606414 on thaps induced astrocytes

By adjusting the concentration of perk inhibitor GSK2606414, the cells were subjected to incubation with Thaps (1.0 μM). After being treated for 24 h, the immunoblotting results revealed that GSK2606414 had the ability to inhibit perk phosphorylation and decrease the expression of ER stress-related proteins in astrocytes (Figure 6).

## 4 Discuss

The incidence of VA is significantly increased after AMI, and VA is closely related to the occurrence of SCD. The occurrence of VA often requires the combined action of abnormal arrhythmogenic structure (matrix) and acute inducement (trigger). More and more evidence show that sympathetic activation is an important factor leading to the occurrence of VA (15). In this study, we first searched the gene bank, used PVN microinjection, cardiac electrophysiology and molecular biology to explore the central mechanism of VA in rats after AMI, and found the following findings: (1) The transcriptome sequencing results of PVN tissue of rats in SH group and AMI group showed that the functions of differentially expressed genes were enriched in protein pathways such as ER stress and neural activity, accompanied by enhanced expression of inflammatory cytokines, and ER stress-related genes were overexpressed; (2) Astrocytes in the PVN in rats were significantly activated and transformed into a pro-inflammatory type after AMI; (3) ER stress in astrocytes of PVN in rats after AMI initiates the UPR through PERK signaling pathway and induces the enhanced expression of inflammatory cytokines; (4) GSK2606414, a selective PERK inhibitor, was found to have no notable impact on astrocyte activation. However, it did demonstrate a significant reduction in the occurrence of VA and improvement in ventricular electrical instability after AMI. These effects are accompanied by a reduction in the activation of central neurons. Based on these findings, it appears that the activation of astrocytes in the PVN of rats with AMI triggers the UPR via the PERK signaling pathway. The above results in increased neuroinflammation and neuronal activity, which leads to increased instability of ventricular electrical activity ultimately.

The normal inflammatory response is transient and protective, which is essential for overall health. However, some inflammatory responses can become excessively activated, leading to cellular dysfunction or tissue damage; If not controlled, it will cause irreversible severe damage and impair the normal inflammatory process that is critical for tissue repair and resolution (16). The delicate balance between beneficial and harmful inflammation has profound implications in the CNS. In the CNS, glial cells, especially astrocytes and microglia, play a key role in controlling the inflammatory environment of the CNS (17, 18). However, in the study of rat model of AMI, it has been found that microglia in the PVN start to be activated only 1–2 weeks following AMI, while no obvious activation was observed 24 h after AMI (19). Therefore, microglia did not have a significant impact on the expression of inflammatory cytokines in the PVN in the early stage of AMI (the first 24 h). In recent years, there has been a growing interest in studying the involvement of astrocytes in inflammation and immune response within the CNS (20–22).

In this strong inflammatory response, cells may cause ER stress due to excessive accumulation of misfolded proteins (23). Although UPR is known to activate pathways related to apoptosis, there is no evidence suggesting that UPR signaling in astrocytes induces cell death. At the same time, it is currently believed that astrocytes activated by UPR have a significant impact on promoting an inflammatory environment in the CNS. Given their abundance and potential harmful effects, astrocytes can contribute to an inflammatory environment in the CNS. They may promote microglial activation and the transport of leukocytes, which can have neurotoxic effects (24). In this experiment, the enhanced expression of ER stress and inflammatory pathways after AMI was verified from the protein levels of the PVN *in vivo* and astrocytes *in vitro*.

When the PERK signal of astrocytes is inhibited, it can yield positive outcomes for cells or animals in general. However, it has no obvious effect on the activation of astrocytes and the pro-inflammatory classification of astrocytes, as indicated by the increased expression of C3 protein. Although A1 astrocytes are neurotoxic *in vitro*, the evidence of A1 astrocytes *in vivo* is limited to the elevation of C3 expression in postmortem samples of various neurodegenerative diseases (25). However, the role of C3 in mediating the pathogenesis is still unclear. Our data support the pathological activation of astrocytes under the action of AMI *in vivo* or tunicamycin *in vitro*. The initiation of UPR to induce central inflammation is the central pathological process. In different diseases (26), astrocytes may have a variety of reactivity, whether there is a certain degree of direct neurotoxicity or not, which may drive the pathogenesis through different mechanisms. Instead of simply categorizing astrocytes as “A1,” “A2,” or “PAN,” it is important to consider the broader range of reactivity they exhibit.

In recent years, astrocytes have become a focal point in the field of CNS research with new discoveries and functions being regularly updated. At present, astrocytes are becoming an important target for the advancement of therapeutic drugs for the CNS (27). According to the available literature and previous research findings, the results of this experiment may be the first discovery that in the early stage of AMI (24 h), the activation of astrocytes in the PVN occurs ER stress. This activation then initiates the UPR via the PERK signaling pathway, leading to central nervous inflammation and excessive sympathetic activity. Ultimately, these factors can impact cardiac electrical stability and contribute to the development of VA. A cytological mechanism of brain heart interaction in the early stage after AMI was elucidated. Considering the role of astrocytes in the PVN, it appears that they could be a promising target within the CNS for preventing and treating VAs following an AMI. Provide a more in-depth the knowledge of astrocytes’ role in the heart-brain-immune axis in AMI and VA development could impact VA management in patients.

### 4.1 Limitations

First of all, the brain nuclei in the CNS and the cell-cell interaction network within the brain nuclei are quite complex. It is not clear whether astrocytes in the PVN will be affected by other nuclei or brain regions, such as the rostral ventrolateral nucleus and the nucleus tractus solitarius, and how the neuronal networks between these nuclei interact to coordinate the activities of the autonomic nervous system; According to the results of gene bank screening and the previous experiments of the research group, we suggest that neuroinflammation alone may be an important factor, and other factors such as neurotransmitter glutamate metabolism may also participate in this network. Second: the number of *in vivo* experimental animals in this experiment is relatively small. it is important to note that the study on the effect of exogenous administration of PERK inhibitor GSK2606414 on AMI model in rats has certain limitations. The study only focused on the impact of the inhibitor without any targeted gene level intervention. Additionally, the study time point for SD rats was limited, which resulted in insufficient comprehensive data. We should thoroughly investigate these issues in our future research.

## 5 Conclusion

At the early stage of AMI (24 h), there is a process called ER stress that occurs in astrocytes in the PVN. This stress triggers a pathway known as PERK-CHOP, which leads to a central inflammatory response and activation of sympathetic neurons. However, researchers have found that inhibiting the phosphorylation of PERK in astrocytes of the PVN can have a significant impact. It can reduce the central inflammatory response and activity of sympathetic neurons in the PVN, ultimately leading to a decrease in sympathetic activation. This reduction in activation can improve the electrical stability of the ventricle during the early stage of AMI and lower the occurrence of VAs.

## Data Availability

The original contributions presented in the study are publicly available. This data can be found here: in the NCBI database sequence read Archive (SRA), accession number srp193705.
